# Supplementary material for: OraSure InteliSwab® Rapid Antigen Test performance with the SARS-CoV-2 Variants of Concern Alpha, Beta, Gamma, Delta, and Omicron
Source: medRxiv. 2022 Feb 4:2022.02.02.22270254. Preprint. [Version 1] doi: 10.1101/2022.02.02.22270254 (PMC8845516; doi:10.1101/2022.02.02.22270254)
Supplement: 1 [file NIHPP2022.02.02.22270254V1-supplement-1.pdf]

medRxiv preprint doi: https://doi.org/10.1101/2022.02.02.22270254; this version posted February 4, 2022. The copyright holder for this preprint (which was not certified by peer review) is the author/funder, who has granted medRxiv a license to display the preprint in perpetuity. This article is a US Government work. It is not subject to copyright under 17 USC 105 and is also made available for use under a CC0 license.

Supplementary Materials:

Table S1: SARS-CoV-2 isolates used in this study

Virus	WHO Description	PANGO Lineage	GiSAID/Genbank Acc
SARS-CoV-2/human/USA/WA-CDC-WA1/2020	Lineage A	WA1	MN985325
England/204820464/2020	Alpha	B.1.1.7	EPI_ISL_683466
USA/MD-HP01542/2021	Beta	B.1.351	EPI_ISL_890360
hCoV-19/USA/GA-EHC-2811C/2021	Gamma	P1	EPI_ISL_7171744
hCoV-19/USA/KY-CDC-2-4242084/2021	Delta	B.1.617.2	EPI_ISL_1823618
hCoV-19/USA/WI-WSLH-221686/2021	Omicron	B.1.1529	EPI_ISL_7263803

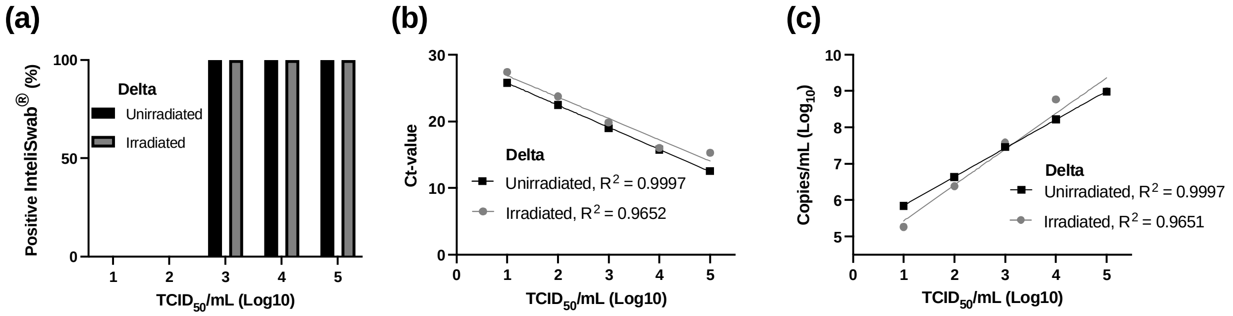

Figure A1. Evaluation of the effects of irradiation on OraSure InteliSwab® results. Two 10-fold dilutions, beginning at 1.00×10^5 $TCID_{50}$/mL, were performed with live Delta and irradiated Delta. For each dilution, 50 µL was applied to 3 separate OraSure InteliSwab®. Irradiation dosage was 2 Mrad. **(a)** Bar plots showing percentage positive OraSure InteliSwab® results for live versus irradiated Delta variant 10-fold dilutions. **(b)** Scatter plot depicting Ct-values for live versus irradiated Delta variant 10-fold dilutions. **(c)** Scatter plot showing RNA copy concentration for live versus irradiated Delta variant 10-fold dilutions.

medRxiv preprint doi: https://doi.org/10.1101/2022.02.02.22270254; this version posted February 4, 2022. The copyright holder for this preprint (which was not certified by peer review) is the author/funder, who has granted medRxiv a license to display the preprint in perpetuity.
This article is a US Government work. It is not subject to copyright under 17 USC 105 and is also made available for use under a CC0 license.

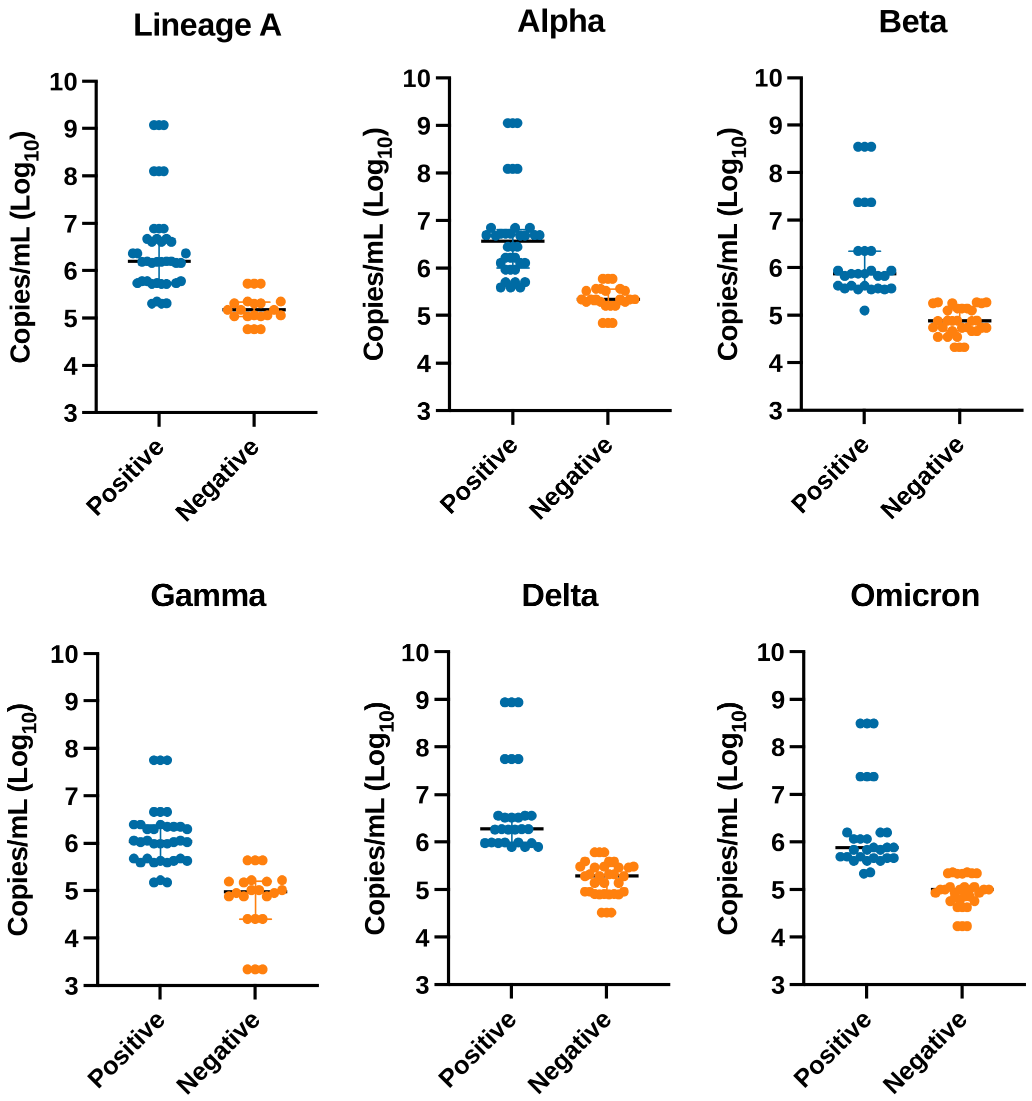

Figure S2. Distribution of all OraSure InteliSwab® results tested on irradiated sample compared to the RNA copies/mL. Scatter plots represent all OraSure InteliSwab® results on irradiated 10-fold and 2-fold dilutions for each variant tested, including lineage A, Alpha, Beta, Gamma, Delta, and Omicron. The blue and orange lines represent the interquartile range for each positive or negative group. The black line represents median.
